# Respiratory health among adolescents living in the Highveld Air Pollution Priority Area in South Africa

**DOI:** 10.1186/s12889-022-14497-8

**Published:** 2022-11-21

**Authors:** Danielle A. Millar, Thandi Kapwata, Zamantimande Kunene, Mirriam Mogotsi, Bianca Wernecke, Rebecca M. Garland, Angela Mathee, Linda Theron, Diane T. Levine, Michael Ungar, Chiara Batini, Catherine John, Caradee Y. Wright

**Affiliations:** 1 Department of Geography, Geoinformatics and Meteorology, University of Pretoria, Pretoria, South Africa; 2 Environment and Health Research Unit, South African Medical Research Council, Johannesburg, South Africa; 3 Department of Environmental Health, Faculty of Health Sciences, University of Johannesburg, Johannesburg, South Africa; 4 Smart Places Cluster, Council for Scientific and Industrial Research, Pretoria, South Africa; 5 Department of Educational Psychology, University of Pretoria, Pretoria, South Africa; 6 Leicester Institute for Advanced Studies, University of Leicester, Leicester, UK; 7 School of Social Work, Dalhousie University, Halifax, Canada; 8 Genetic Epidemiology Group, Department of Health Sciences, University of Leicester, Leicester, UK; 9 Department of Health Sciences, University of Leicester, Leicester, UK; 10 Environment and Health Research Unit, South African Medical Research Council, Pretoria, South Africa

**Keywords:** Air quality management, Air pollution, Environmental health, Environmental pollution, Industrial emissions, Public health

## Abstract

**Background:**

Air pollution is a global, public health emergency. The effect of living in areas with very poor air quality on adolescents’ physical health is largely unknown. The aim of this study was to investigate the prevalence of adverse respiratory health outcomes among adolescents living in a known air pollution hotspot in South Africa.

**Methods:**

Ambient air quality data from 2005 to 2019 for the two areas, Secunda and eMbalenhle, in the Highveld Air Pollution Priority Area in Mpumalanga province, South Africa were gathered and compared against national ambient air pollution standards and the World Health Organization Air Quality Guidelines. In 2019, adolescents attending schools in the areas completed a self-administered questionnaire investigating individual demographics, socio-economic status, health, medical history, and fuel type used in homes. Respiratory health illnesses assessed were doctor-diagnosed hay fever, allergies, frequent cough, wheezing, bronchitis, pneumonia and asthma. The relationship between presence (at least one) or absence (none) of self-reported respiratory illness and risk factors, e.g., fuel use at home, was explored. Logistic regression was used to estimate the odds ratio and 95% confidence interval (CI) of risk factors associated with respiratory illness adjusted for body mass index (measured by field assistants), gender, education level of both parents / guardians and socio-economic status.

**Results:**

Particulate matter and ozone were the two pollutants most frequently exceeding national annual air quality standards in the study area. All 233 adolescent participants were between 13 and 17 years of age. Prevalence of self-reported respiratory symptoms among the participants ranged from 2% for ‘ever’ doctor-diagnosed bronchitis and pneumonia to 42% ever experiencing allergies; wheezing chest was the second most reported symptom (39%). Half (52%) of the adolescents who had respiratory illness were exposed to environmental tobacco smoke in the dwelling. There was a statistically significant difference between the presence or absence of self-reported respiratory illness based on the number of years lived in Secunda or eMbalenhle (*p* = 0.02). For a one-unit change in the number of years lived in an area, the odds of reporting a respiratory illness increased by a factor of 1.08 (*p* = 0.025, 95% CI = 1.01–1.16). This association was still statistically significant when the model was adjusted for confounders (*p* = 0.037).

**Conclusions:**

Adolescents living in air polluted areas experience adverse health impacts Future research should interrogate long-term exposure and health outcomes among adolescents living in the air polluted environment.

**Supplementary Information:**

The online version contains supplementary material available at 10.1186/s12889-022-14497-8.

## Introduction

Air pollution has long been a major environmental risk to human health [1]. Exposure to both outdoor and indoor air pollution can cause a wide range of respiratory illnesses, such as chronic obstructive pulmonary disease, asthma, bronchiolitis, wheezing, shortness of breath, and lung cancer [2, 3]. Long-term exposure to air pollution has also been found to be associated with cardiovascular diseases, nervous system dysfunctions, and cutaneous diseases [4–6].

It is estimated that around 91% of the world’s population reside in places where concentrations of air pollutants exceed the World Health Organization (WHO) recommended guidelines [7]. Pollutants of importance include particulate matter with an aerodynamic diameter of less than 2.5 μm (PM_2.5_) and less than 10 μm (PM_10_), ozone (O_3_), nitrogen dioxide (NO_2_), and sulphur dioxide (SO_2_).

The WHO provides recommended guidelines for these pollutants aimed to protect human health; for example, for PM_2.5_ and PM_10_ the guideline values are 10 μg/m^3^ and 20 μg/m^3^, respectively [7]. The South African National Ambient Air Quality Standards (NAAQS) [8] are less stringent than the WHO guidelines (see Supplementary Table S1). Given the South African climate and diverse natural activities such as veld fires, particularly in the semi-arid Highveld region which experiences dry winter months, the guideline for PM_2.5_ values would not be realistic in the South African setting [9].

Outdoor pollution is mostly attributed to vehicles, power generation, building heating systems, agriculture / waste incineration, and industry, as well as natural sources such as pollen, windblown dust, plumes from volcanic eruptions and sea salt spray [7]. People in low- or middle-income countries may be reliant on highly-polluting solid fuels such as wood, coal, animal dung, and crop wastes for heating, cooking, and lighting, thereby causing indoor air pollution and increasing the air pollution-related disease burden [10–12].

In November 2007, the Mpumalanga Highveld of South Africa was declared part of the Highveld Air Pollution Priority Area (HPA) by the South African government [13] since ambient air quality in major towns such as eMalahleni, Middelburg, Secunda, Standerton, Edenvale, Boksburg, and Benoni exceeded or were likely to exceed the South African National Ambient Air Quality Standards and that the area requires specific air quality management action to rectify the situation [13]. Satellite data suggests that Mpumalanga is one of the world’s largest air pollution hotspots, with sources including emissions being coal-fired power stations, petrochemical plants, metal smelters, and mines [9, 14, 15]. People living and working in these areas are exposed to air quality that is potentially harmful to their health and well-being [16, 17].

Children and adolescents are particularly vulnerable to environmental risks such as air pollution exposure, even at low levels, due to their developing organs such as lungs and the brain, immune system and metabolic functions, and time spent outdoors, e.g., walking to school or playing sport [18, 19]. Also, children inhale a higher volume of air per body weight than adults [20]. Evidence suggests that PM_2.5_, PM_10_, O_3_ and NO_2_ are associated with respiratory diseases such as asthma, lung function deficits and air way inflammation in children [19, 21]. However, there is limited research of potential relations between air quality and related respiratory health outcomes among adolescents in South Africa [22–24].

The aim of the study was to understand adolescent respiratory health and associated risk factors among adolescents living in areas characterized by poor ambient air quality in the HPA. The aim was supported by two research objectives: 1) to determine the prevalence of adverse respiratory health outcomes among adolescents living in an air pollution hotspot; and 2) to consider household-related risk factors related to adverse respiratory health outcomes in the adolescents.

## Methods

### Study population

**Fig. 1:**
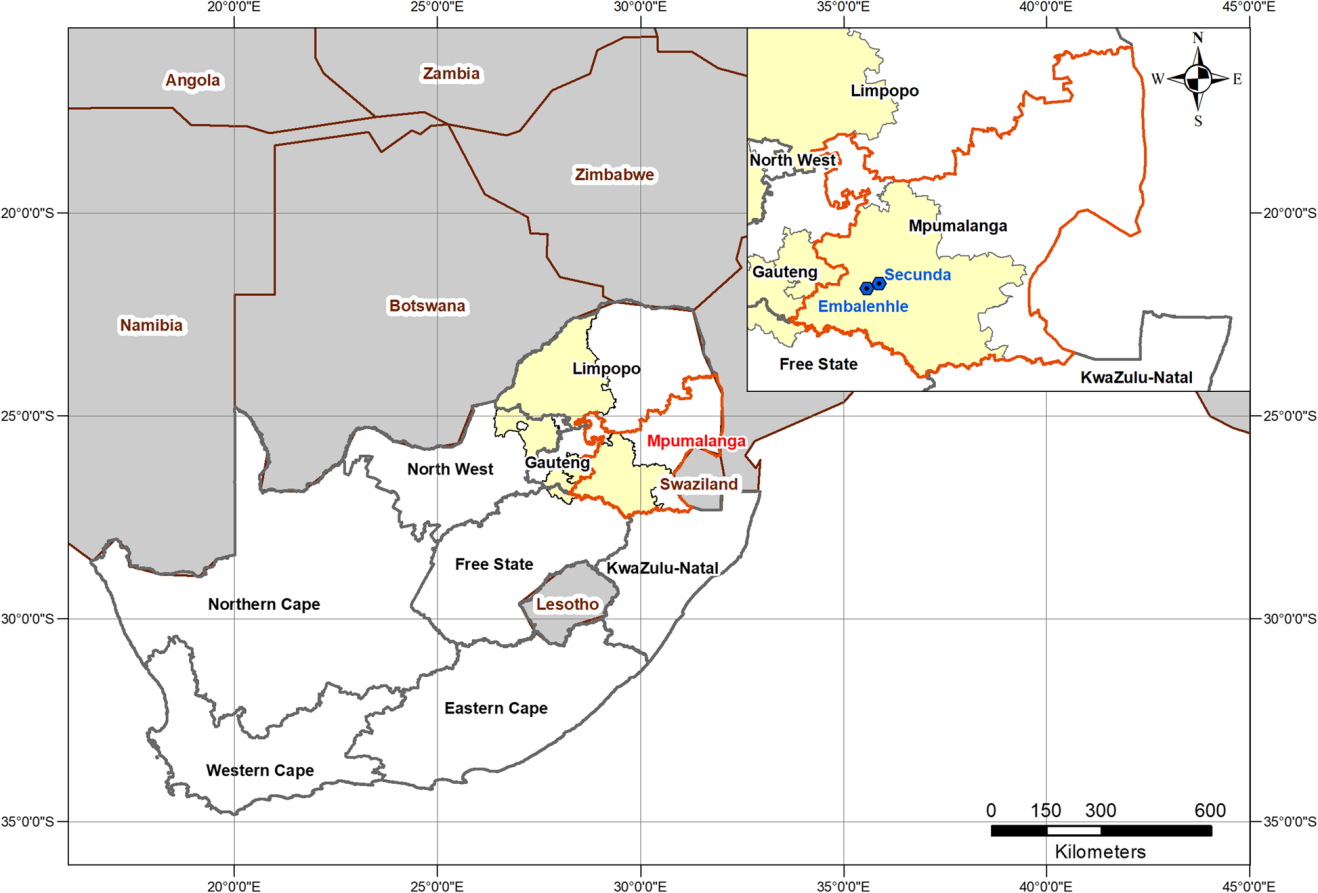
Map illustrating the study sites in Mpumalanga province, South Africa. Highveld Air Pollution Priority Area shown in yellow

The study took place in Mpumalanga Province, South Africa, in the areas of Secunda and the adjacent eMbalenhle which are 17.3 km apart (Fig. 1). The town of Secunda has approximately 40,000 residents; (231 persons/km^2^) and the adjacent community eMbalenhle has approximately 119,000 residents (6050 persons/km^2^); some 822 people per km^2^. They are in close proximity to coal-fired power stations, mines, and a coal liquefaction plant. The Govan Mbeki District Municipality, which includes Secunda and eMbalenhle, reported approximately 26.2% unemployment in 2011 [25]. This percentage was higher than the national unemployment of 25% in 2011 [25].

### Ambient air quality assessment

To give a context to the air pollution situation in the study area, ambient air quality data between 2005 and 2019 at three ambient air quality monitoring stations (Table 1) were analysed. One of the stations was in Secunda and two stations were in eMbalenhle. Data were downloaded from the South African Air Quality Information System (SAAQIS) [26] and then underwent quality control procedures.

**Table 1:** Ambient air quality monitoring stations in Mpumalanga Province from which air quality data were downloaded via SAAQIS for analyses. Timeframes for which data were considered are inconsistent due to the different data availability from the sites

Station (Custodian of the station)	Co-ordinates	Type	Timeframe for which data were considered	Pollutants considered
Secunda Club Station (Sasol)	- 26.523518 29.189015	Residential – Middle-to-high income	2005–2019	PM_2.5_, PM_10_, SO_2_, NO_2_, O_3_
Embalenhle Station (SAWS)	−26.550639 29.079028	Residential – Low income	2008–2019	PM_2.5_, PM_10_, SO_2_, NO_2_
Embalenhle Station (Sasol)	−26.551427 29.112381	Residential – Low income	2016–2019	PM_2.5_, PM_10_, SO_2_, NO_2_, O_3_

*DFFE* Department of Forestry, Fisheries and Environment

The primary pollutants PM_2.5_, PM_10_, SO_2_, NO_2_ and O_3_, were considered for compliance with their respective NAAQS (Supplementary Table S1). Compliance to hourly (SO_2_ and NO_2_) and daily (PM_2.5_, PM_10_, and SO_2_) standards were assessed by determining the frequency of exceedances of the relevant ambient standard limit value (e.g., four allowed exceedances for daily NAAQS and 88 allowed exceedances for the hourly NAAQS) using the 99th percentile.

A data availability threshold of 75% was applied for the calculation of the averaging periods. For this study and for comparison purposes, the current applicable PM_10_ and PM_2.5_ standards were used for all considered years to determine compliance. These standards are the strictest standards South Africa has imposed. Years without data or years that did not pass the data completeness threshold of 75% were considered missing data.

Other Secunda / eMbalenhle monitoring sites (eMbalenhle North and eMbalenhle South on SAAQIS) were excluded from this analysis as they only provided data from ~ 2017 (less than three years) and longer time series were required for this study. Due to the varying levels of data completeness across years, direct comparisons across years for any of the values is not possible.

### Data collection

Adolescents attending four schools in Secunda and eMbalenhle in Grades eight to ten who had lived in the study areas for more than 12 months were recruited for the study. Invited schools were from a list of local schools provided to the researchers by a study team working in the same area to prevent overlap and working in the same schools. Principals of the schools were contacted and their consent to perform the study in their schools was obtained.

Adolescents were given the opportunity to accept or decline the invitation to participate. A questionnaire self-completed by the students was used to collect information regarding demographics, socio-economic status, health, medical history and fuel type used in homes. Health outcomes were also considered in this questionnaire (Supplementary Tables S2 – S6). The survey was electronic and was completed on a tablet using Redcap software [27]; a secure web application used to conduct surveys, questionnaires and to manage databases.

### Health outcomes

To meet objective 1, respiratory health illnesses included in the questionnaire were hay fever, allergies (i.e., stuffy / itchy / runny nose and watery / itchy eyes), frequent cough, wheezing, bronchitis, pneumonia, and asthma. These symptoms are in keeping with the ISAAC questionnaire. Positive responses were coded as 1 and negative responses were coded as 0.

### Risk factors

To meet objective 2, the following were included in the questionnaire: demographics [age, biological sex], socio-economic status [main source of income in the household, education level of both parents/caregivers]; presence or absence of environmental tobacco smoke or pets in the dwelling; duration of time lived in area; and fuel type used in homes [type of fuel (electricity, gas, wood, coal, paraffin, oil, other) used for cooking / heating]. Risk factors were chosen a priori based on literature.

### Statistical analyses

Statistical analyses were performed using STATA version 15 [28]. The Pearson chi-square test and Independent t-test were used to determine the relationship between the presence (at least one) or absence (none) of self-reported respiratory illness and categorical and continuous risk factors. Univariable and multivariable Logistic regression were used to estimate the odds ratio (OR) and 95% confidence interval (CI) of risk factors associated with respiratory illness. The risk factor environmental tobacco smoke (ETS) was computed from positive responses for questions relating to whether a parent / guardian or any other family member that smoked in the home. Models were adjusted for following potential confounding factors: age, body mass index (BMI), using height and weight data collected by field assistants, biological sex, education level of both parents / guardians, and socio-economic status. These were selected because previous research has shown that these factors may exacerbate health outcomes and exposure related to air pollution [29–31]. Analyses were done for participants from Secunda and eMbalenhle combined since eMbalenhle constitutes an area of the greater town of Secunda. The final multivariable logistic regression model was evaluated using the Hosmer-Lemeshow goodness of fit test.

### Research ethics

Permission was obtained from the Provincial Department of Education as well as the School Principals. The protocol for recruitment, data, and sample collection for the study was approved by the University of Pretoria Research Ethics Committee (22 July 2019, UP17/05/01). Signed informed consent was obtained from the participants’ guardian / caregiver, as well as signed assent from the participants. Only with the approval of both consent and assent did an adolescents participate in the study. Participation was strictly voluntary and determined by the adolescent’s willingness to participate.

## Results

### Ambient air quality findings

Ambient data were analysed to consider hourly, daily and annual concentrations and findings are given in the Supplementary Files. The annual mean concentrations of the five major pollutants (i.e., PM_2.5_, PM_10_, SO_2,_ NO_2_, and O_3_) are summarized in Tables 2, 3 and 4. Between 2005 and 2019, the annual PM_10_ NAAQS (the ‘new’ standard that came into effect on 1 January 2015) was exceeded only once in 2014 at the Club Station in Secunda (Table 3). For the remaining years, the annual average PM_10_ concentrations were very close to the 40 μg/m^3^ limit value threshold, but did not exceed it. No other pollutants exceeded the annual NAAQS at Secunda for this period.

**Table 2:** Annual mean levels for five air pollutants measured at Secunda Club Station (Sasol) for 2005–2019 (NAAQS exceedances highlighted in bold font; years without data or years that did not pass the data completeness threshold of 75% denoted with “--“)

**Pollutant**	**NAAQS limit**	**2005**	**2006**	**2007**	**2008**	**2009**
**PM**_**2.5**_	20 μg/m^3^	–	–	–	–	–
**PM**_**10**_	40 μg/m^3^	38	35	39	37	–
**SO**_**2**_	19 ppb	9	8	6	–	8
**NO**_**2**_	21 ppb	7	–	11	11	8
**Pollutant**	**NAAQS limit**	**2010**	**2011**	**2012**	**2013**	**2014**
**PM**_**2.5**_	20 μg/m^3^	–	–	13	13	15
**PM**_**10**_	40 μg/m^3^	30	28	30	33	**45**
**SO**_**2**_	19 ppb	7	7	8	7	9
**NO**_**2**_	21 ppb	10	10	9	11	14
**Pollutant**	**NAAQS limit**	**2015**	**2016**	**2017**	**2018**	**2019**
**PM**_**2.5**_	20 μg/m^3^	–	16	–	–	–
**PM**_**10**_	40 μg/m^3^	–	38	–	–	–
**SO**_**2**_	19 ppb	7	8	–	–	–
**NO**_**2**_	21 ppb	11	9	–	–	–

**Table 3:** Annual mean levels for five air pollutants measured at Embalenhle (SAWS) for 2008–2019

**Pollutant**	**NAAQS limit**	**2005**	**2006**	**2007**	**2008**	**2009**
**PM**_**2.5**_	20 μg/m^3^	–	–	–	–	**38**
**PM**_**10**_	40 μg/m^3^	–	–	–	–	**72**
**SO**_**2**_	19 ppb	–	–	–	–	8
**NO**_**2**_	21 ppb	–	–	–	–	–
**Pollutant**	**NAAQS limit**	**2010**	**2011**	**2012**	**2013**	**2014**
**PM**_**2.5**_	20 μg/m^3^	**38**	–	**27**	**29**	–
**PM**_**10**_	40 μg/m^3^	**87**	–	**61**	**71**	–
**SO**_**2**_	19 ppb	11	–	10	–	10
**NO**_**2**_	21 ppb	–	–	6	12	14
**Pollutant**	**NAAQS limit**	**2015**	**2016**	**2017**	**2018**	**2019**
**PM**_**2.5**_	20 μg/m^3^	–	–	–	–	–
**PM**_**10**_	40 μg/m^3^	–	–	–	–	–
**SO**_**2**_	19 ppb	–	6	4	1	4
**NO**_**2**_	21 ppb	–	–	–	–	–

**Table 4:** Annual mean levels for five air pollutants measured at Embalenhle (Sasol) for 2008–2019

**Pollutant**	**NAAQS limit**	**2005**	**2006**	**2007**	**2008**	**2009**
**PM**_**2.5**_	20 μg/m^3^	–	–	–	–	–
**PM**_**10**_	40 μg/m^3^	–	–	–	–	–
**SO**_**2**_	19 ppb	–	–	–	–	–
**NO**_**2**_	21 ppb	–	–	–	–	–
**Pollutant**	**NAAQS limit**	**2010**	**2011**	**2012**	**2013**	**2014**
**PM**_**2.5**_	20 μg/m^3^	–	–	–	–	–
**PM**_**10**_	40 μg/m^3^	–	–	–	–	–
**SO**_**2**_	19 ppb	–	–	–	–	–
**NO**_**2**_	21 ppb	–	–	–	–	–
**Pollutant**	**NAAQS limit**	**2015**	**2016**	**2017**	**2018**	**2019**
**PM**_**2.5**_	20 μg/m^3^	–	**20**	**21**	–	–
**PM**_**10**_	40 μg/m^3^	–	**50**	**53**	–	–
**SO**_**2**_	19 ppb	–	7	6	–	–
**NO**_**2**_	21 ppb	–	–	12	–	–

Annual PM_2.5_ and PM_10_ averages measured at the Embalenhle (SAWS) ambient monitoring station between 2009 and 2019 exceeded their respective NAAQS for each year for which the 75% data availability threshold was met (Table 3). No other pollutants exceeded the annual NAAQS at the Embalenhle (SAWS) station for this period.

Data at the Embalenhle (Sasol) monitoring site were only available for two years (2016–2017) (Table 4). Annual PM_2.5_ and PM_10_ NAAQS were exceeded in these two years. Compliance with the annual SO_2_ and NO_2_ NAAQS was noted when data were available.

The 99th percentile 8-hour running average NAAQS for O_3_ was exceeded every year for which the average could be calculated at both Club Station and at Embalenhle (Sasol) (Tables 5 and 6). The O_3_ data at the Embalenhle (SAWS) site were not included in the analysis, as the data did not pass data quality and quantity requirements.

**Table 5:** The 99th percentile 8-hour running average for ozone (8-hourly running average) measured at Club Station, Secunda

**Pollutant**	**NAAQS limit**	**2005**	**2006**	**2007**	**2008**	**2009**
**O**_**3**_	61 ppb	**82**	**76**	**68**	**107**	**75**
**Pollutant**	**NAAQS limit**	**2010**	**2011**	**2012**	**2013**	**2014**
**O**_**3**_	61 ppb	**64**	**75**	**70**	**169**	**83**
**Pollutant**	**NAAQS limit**	**2015**	**2016**	**2017**	**2018**	**2019**
**O**_**3**_	61 ppb	**72**	**200**	**62**	–	**70**

**Table 6:** The 99th percentile 8-hour running average for ozone (8-hourly running average) measured at Embalenhle (Sasol)

**Pollutant**	**NAAQS limit**	**2005**	**2006**	**2007**	**2008**	**2009**
**O**_**3**_	61 ppb	–	–	–	–	**–**
**Pollutant**	**NAAQS limit**	**2010**	**2011**	**2012**	**2013**	**2014**
**O**_**3**_	61 ppb	–	–	–	–	**–**
**Pollutant**	**NAAQS limit**	**2015**	**2016**	**2017**	**2018**	**2019**
**O**_**3**_	61 ppb	–	**71**	**70**	**68**	**72**

In the Supplementary Tables, compliance with the hourly and daily NAAQS for the various pollutants are presented (Tables S7, S8, and S9). Exceedances of the respective standards are highlighted in bold. The daily PM_10_ and PM_2.5_ NAAQS were almost consistently exceeded at all sites for all years for which data were available. Also, the PM_2.5_ and PM_10_ annual mean concentrations exceeded the WHO recommended guidelines every year for which data were available and analysed.

The daily NAAQS for SO_2_ was exceeded once (at the Secunda DFFE station in 2012). The hourly NAAQS for NO_2_ was exceeded on three occasions (2007 at Club Station (Secunda), 2018 in Embalenhle (SAWS), and 2016 at Embalenhle (Sasol)).

### Respiratory health outcomes and associated risk factors

A total of 233 adolescents aged between 13 and 17 years were recruited from four secondary schools. Most adolescents were aged between 13 and 15 years (97%) and were predominantly female (72%) (Table 7). About a third of adolescents’ households were dependent on social grants as the primary source of income.

**Table 7:** Descriptive profile of the sample population of adolescents in the study

Variable (Sample ***N*** = 233)	Frequency	
	***n***	%
Town		
Secunda	33	14
Embalenhle	200	86
Gender of the participant		
Male	65	28
Female	167	72
*Missing*	*1*	*0*
Age of the participant		
13	49	22
14	90	41
15	76	34
16	6	3
17	1	< 1
*Missing*	*11*	*4*
Fuel type usually used for cooking		
Electricity	207	91
Gas	14	6
Wood	1	> 1
Coal	4	2
Other	2	> 1
*Missing*	*5*	*2*
Fuel type usually used for heating		
Electricity	159	69
Gas	18	8
Paraffin	6	3
Wood	17	7
Coal	26	11
Oil	5	2
Other	–	–
*Missing*	*2*	*0*
Household income		
Salaries/commission	102	45
Income from business	26	11
Maintenance	7	3
Pension	7	3
Social grant	75	33
Sales of farm produce/services	1	> 1
Other	*11*	*5*
Presence of environmental tobacco smoke in the home		
No	112	48
Yes	121	52

Prevalence of self-reported and doctor-diagnosed respiratory symptoms among the participants ranged from 2% for an ‘ever’ doctor-diagnosed bronchitis and pneumonia to 42% ever experiencing allergies (Table 8). Wheezing chest was the second most reported respiratory symptom among participants (39%). Half of the adolescents who had respiratory illness were exposed to environmental tobacco smoke (52%), due to a parent / guardian, the participant, or any other family member smoking in the dwelling.

**Table 8:** Prevalence of self-reported and self-reported, doctor-diagnosed respiratory symptoms among adolescents from Secunda and eMbalenhle (combined)

Type of diagnosis	Respiratory health outcome	***N*** (%)	
		Yes	No
Self-reported			
Ever experienced	Allergies	96 (42)	132 (58)
	Frequent cough	54 (23)	178 (76)
	Wheezing chest	86 (39)	137 (61)
Self-reported, doctor-diagnosed			
Ever experienced	Bronchitis	4 (2)	223 (98)
	Pneumonia	5 (2)	223 (98)
	Asthma	18 (8)	215 (92)
	Hay fever	48 (21)	181 (79)

The independent t-test results (Table 9) showed that there was a statistically significant difference between the presence or absence of self-reported respiratory illness based on the number of years lived in Secunda or eMbalenhle (*p* = 0.02). The mean number of years lived in the area was slightly higher for children who reported respiratory illness (12 compared to 10 years).

**Table 9:** Characteristics of adolescents’ self-reported living conditions related to air pollution exposure and presence/absence of respiratory illness

		Overall frequency ***N*** (%)	Model results		***p***-value
			Any respiratory illness n (%)	No respiratory illness ***n*** (%)	
Does the adolescent smoke	No*	226 (98)	182 (99)	44 (98)	0.55
	Yes	3 (1)	2 (1)	1 (2)	
Presence of environmental tobacco smoke in the home	No*	113 (48)	88 (47)	25 (53)	0.47
	Yes	120 (52)	98 (53)	22 (47)	
Main fuel used for cooking	Electric*	207 (90)	130 (71)	29 (62)	0.31
	Non-electric	21 (9)	54 (29)	18 (38)	
Main fuel used for heating	Electric*	159 (68)	167 (92)	40 (87)	0.24
	Non-electric	72 (31)	15 (8)	6 (13)	
			**Mean (min - max)**	**Mean (min - max)**	
Number of household members	–	–	5 (1–16)	5 (2–12)	0.94
Number of years living in Secunda or eMbalenhle	–	–	12 (1–16)	10 (1–16)	0.022

Note. * indicates the reference category for each variable in the analysis

**Fig. 2:**
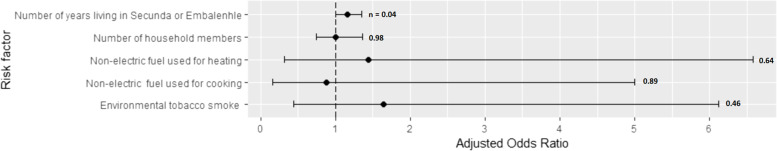
Logistic regression results indicating risk factors for respiratory illnesses among the adolescents in the study population. Final model omitted respondent smoking as a risk factor due to a dependency among the independent variables

Number of years living in Secunda or eMbalenhle was the only statistically significant risk factor in the univariable regression analysis (Fig. 2). For a one-unit change in the number of years lived in an area, the odds of a respiratory illness increased by a factor of 1.09 (*p* = 0.018, 95% CI = 1.01–1.16). This association was still statistically significant in the multivariable analysis when the model was adjusted for confounders (OR = 1.16, *p* = 0.039, 95% CI = 1.01–1.35). The confounders included age, BMI category, level of parent education (separate variables for mother and father), and socio-economic status (primary source of household income). Hosmer-Lemeshow goodness of fit test was *P* = 0.57 indicating that the model was a good fit.

## Discussion

Our study considered the impact of air pollution on adolescent health in a highly air polluted area in South Africa. While we did not find strong evidence for associations between air pollution and respiratory health; a small effect was possible for duration of living in a polluted environment. This may also have been influenced by the relatively small sample size and that we merged towns that likely have different characteristics.

We found that adolescents lived in households that mainly relied on electricity for cooking and heating, although about 10% of adolescents reported that coal was used for heating. Half of the adolescents reported the presence of environmental tobacco smoke in the home. Evidence suggests that exposure to environmental tobacco smoke is associated with childhood upper and lower respiratory tract infection, wheezing and asthma [32]. A Chinese study found that indoor tobacco smoke and respiratory illness (pneumonia, common cold, croup and dry night cough) were significantly associated in children aged 3–8 years with ORs ranging from 1.06 to 1.95 [33]. An increased frequency in asthma was found in high school students aged 11–16 years exposed to environmental tobacco smoke (OR = 1.08, 95% CI, 1.05–1.12) and cigarette smoking (OR = 1.29, 95% CI, 1.17–1.42) in Taiwan [34]. A study in Greece showed that adolescents aged 13–15 years living with mothers who smoke had double the exhaled carbon monoxide compared to non-smoking families [35].

The most common self-reported respiratory health outcomes were allergies, frequent cough, wheezing chest, and doctor-diagnosed hay fever. All of these outcomes have been associated with exposure to air pollution [36–40]. Regarding hay fever, the timing of the study is an important consideration. Most respondents were recruited in September (56%), October (34%) and November (10%) which are austral spring months. These months are associated with high pollen counts in Mpumalanga [41] and this may have influenced the proportion of adolescents reporting allergies and hay fever.

Ambient air pollution levels in the study area were generally above national and international limits, specifically for PM_2.5_, PM_10_ and O_3_. This was unsurprising given the location of the two study areas in the HPA. Previous studies have found similar air pollution levels in HPA [17, 42]. Although a quantitative comparison between the sites, it is important to note that the Embalenhle (SAWS) and Embalenhle (Sasol) sites had exceedances of the NAAQS for PM daily and annually for every time data was available, except the Embalenhle (Sasol) PM_10_ averages in 2019, compared to Club, which did not have as many exceedances.

A statistically significant association between the number of years that the adolescent had lived in the area (both areas combined) and the presence of respiratory illnesses was found. Given the ambient air quality findings for the study area, particularly PM and O_3_, that exceeded limits set to protect human health, this association is reason for concern.

While ambient air quality management is a definitely a priority for this area, primary interventions may also be helpful. Ensuring regular waste collection to prevent waste burning and increasing household income, especially in a study area such as ours where reliance on social grants was relatively high, have been shown to potentially be important for people living in Embalenhle [43].

While this study sample was relatively small, it is the first of its kind to consider self-reported adolescent respiratory health among individuals living in the highly polluted HPA and thus provides valuable baseline data. Some limitations were identified. Schools were not randomly selected, however, are considered to be representative of the schools in the area. Moreover, the small sample size contributed to the risk of underreporting. The adolescent survey asked for ‘ever’ occurrence of respiratory health outcomes, rather than ‘in the past two weeks or past year’. This may have led to over-representation of occurrence, but we deemed this a more reliable measure than asking young people to recall different time periods. We did not ask about certain dwelling characteristics that may also influence household air pollution exposure, which could have contributed to exposure misclassification. For example, ventilation and roof type should be included in more detailed assessments of household air pollution exposure. Our study was conducted during spring; had the study been conducted during winter different fuel sources for heating and cooking may have been given by the adolescents. Household fuel use patterns are known to differ by season [44] hence a repeated cross-sectional or long-term study of fuel use patterns in different seasons would be important for future studies.

Our study provides information on self-reported health outcomes among individuals living in a highly polluted environment in a peri-urban environment for adolescents. Given the specific air pollution concerns for the area, peri-urban nature of the location, and the small sample size, these results cannot be generalised to urban or rural communities, or to children of other age groups. Similar studies in communities in different settings, and involving adolescents and children of different age groups, would be of considerable interest. There is a need for interdisciplinary research that speaks to intersections between multiple systems [45] including the natural environment, physical health, and the uniqueness of both the South African context and adolescence as a life stage.

## Conclusions

Our results suggest that adolescents living in areas located within the HPA are adversely affected by air pollution, in particular O_3_ and PM. It is essential that we work towards meeting the NAAQS in this area to protect adolescent health. Future research should investigate long-term exposure and health outcomes among adolescents living in the HPA given the risk factor findings here.

## Supplementary Information

**Additional file 1.**

## Data Availability

The datasets analysed during the current study are available from the corresponding author on reasonable request.

## Abbreviations

AQG: Air Quality Guidelines
BMI: Body Mass Index
CI: Confidence Interval
DFFE: Department of Forestry, Fisheries and Environment
HPA: Highveld Priority Area
IT: Inter Target
NAASQ: South African National Ambient Air Quality Standards
NO_2_: Nitrogen Dioxide
O_3_: Ozone
OR: Odds Ratio
PM_2.5_: Particulate Matter with an Aerodynamic Diameter of Less Than 2.5 Micrometres
PM_10_: Particulate Matter with an Aerodynamic Diameter of Less Than 10 Micrometres Value
SAAQIS: South African Air Quality Information System
SO_2_: Sulphur Dioxide
WHO: World Health Organization
