# Supplementary material for: Respiratory health among adolescents living in the Highveld Air Pollution Priority Area in South Africa
Source: BMC Public Health. 2022 Nov 21;22:2136. doi: 10.1186/s12889-022-14497-8 (PMC9677637; doi:10.1186/s12889-022-14497-8)
Supplement: Supplementary file 1 — Additional file 1. [file 12889_2022_14497_MOESM1_ESM.docx]

Supplementary Tables

**Table S1.** Overview of South African National Ambient Air Quality Standards and the WHO Air Quality Guidelines (WHO 2021, NAAQS 2009, NAAQS 2012).

|  |  |  | **South African National Ambient Air Quality Standards** | | **WHO Guidelines Interim Targets (IT) and Air Quality Guideline (AQG)** | | | | |
| --- | --- | --- | --- | --- | --- | --- | --- | --- | --- |
| **Pollutant** | **Averaging time** | **Unit** | **Current** | **1 Jan 2030** | **IT-1** | **IT-2** | **IT-3** | **IT-4** | **AQG** |
| PM_2.5_ | 24-hour | µg/m^3^ | 40 ^a^ | 25 | 75^1^ | 50 | 37.5 | 25 | 15 |
|  | 1-year | µg/m^3^ | 20 | 15 | 35 | 25 | 15 | 10 | 5 |
| PM_10_ | 24-hour | µg/m^3^ | 75 ^a^ | n.c. | 150^1^ | 100 | 75 | 50 | 45 |
|  | 1-year | µg/m^3^ | 40 | n.c. | 70 | 50 | 30 | 20 | 15 |
| O_3_ | 8-hour | µg/m^3^ | 120 ^b^ | n.c. | 160^1^ | 120 | - | - | 100 |
|  | Peak season ^2^ | µg/m^3^ | - | - | 100 | 70 | - | - | 60 |
| SO_2_ | 10 minutes | µg/m^3^ | 500^d^ | n.c. | - | - | - | - | - |
|  | 1 hour | µg/m^3^ | 350^e^ | n.c. |  |  |  |  |  |
|  | 24 hours | µg/m^3^ | 125^a^ | n.c. | 125^1^ | 50 | - | - | 40 |
|  | 1 year | µg/m^3^ | 50 | n.c. | - | - | - | - | - |
| NO_2_ | 1 hour | µg/m^3^ | 200^e^ | n.c. | - | - | - | - | - |
|  | 24 hours | µg/m^3^ | - | None | 120^1^ | 50 | - | - | 25 |
|  | 1 year | µg/m^3^ | 40 | n.c. | 40^1^ | 30 | 20 | - | 10 |
| CO | 1 hour | mg/m^3^ | 30^e^ | n.c. |  |  |  |  |  |
|  | 8 hour (calculated on 1 hourly averages) | mg/m^3^ | 10^b^ | n.c. |  |  |  |  |  |
|  | 24 hours | µg/m^3^ | - | - | 7^1^ | - | - | - | 4 |

Notes: ^a^ Allowed number of exceedances = 4 / yr, ^b^ Allowed number of exceedances = 11 / yr; ^c^ Allowed number of exceedances = 3 / yr; ^d^ Allowed number of exceedances = 526 / yr; ^e^ Allowed number of exceedances = 88 / yr; ^1^ 99^th^ percentile, i.e. 3-4 exceedances per year; ^2^ Average of daily maximum 8-hour mean O₃ concentration in six consecutive months with the highest 6-month running average O₃ concentration.; n.c. = no change; NA = no interim target available

**Table S2:** Questions pertaining to socio-economic demographic characteristics as presented in the self-administered questionnaire.

| **What is your gender**  Male  Female |
| --- |
| **Which language do you speak at home?**  Zulu  Siswati  Setswana  Sesotho  Sepedi  Xitsonga  Tshivenda  English  Afrikaans  Sindebele  Other |
| **Were you born in South Africa?**  Yes  No |
| **Who do you live with at home?**  My father  My mother  My step-mum  My step-dad  Friends  Siblings  Grandparent(s)  Foster parents  Other |
| **What is your everyday main source of income at home? Or the one that brings in the most money? (Please choose one)**  Salaries/commission  Income from a business  Maintenance  Pensions  Social grants  Sales of farm products and services  Other |
| **What level of education has your mother/stepmother/female guardian received?**  None  Primary  Secondary  Tertiary  Don’t know |
| **What level of education has your father/stepfather/male guardian received?**  None  Primary  Secondary  Tertiary  Don’t know |

**Table S3:** Questions pertaining to household/dwelling characteristics as presented in the self-administered questionnaire.

| **In your house, what fuel is usually used to cook?**  Electricity  Gas  Wood  Coal  Other |
| --- |
| **In your house, what fuel is usually used for heating?**  Electricity  Gas  Paraffin  Wood  Coal  Oil  Other |
| **How often do trucks pass through the street where you live?**  Never  Seldom  Frequently through the day  Almost the whole day |
| **In the past 12 months, have you had a cat in your home?**  Yes  No |
| **In the past 12 months, have you had a dog in your home?**  Yes  No |
| **Does your father/stepfather/male guardian smoke cigarettes?**  Yes  No |
| **Does your mother/stepmother/female guardian smoke cigarettes?**  Yes  No |

**Table S4:** Questions pertaining to nutrition as presented in the self-administered questionnaire.

| **In the past 12 months, how often, on average, did you eat or drink the following? (Please leave blank if you do not know what the food is)**  **Meat (e.g. chicken, beef)**  Never or occasionally  Once or twice a week  Three or more times a week  **Seafood (e.g. fish)**  Never or occasionally  Once or twice a week  Three or more times a week  **Fruit**  Never or occasionally  Once or twice a week  Three or more times a week  **Vegetables (green and root)**  Never or occasionally  Once or twice a week  Three or more times a week  **Grains**  Never or occasionally  Once or twice a week  Three or more times a week  **Cereal (including bread)**  Never or occasionally  Once or twice a week  Three or more times a week  **Pasta**  Never or occasionally  Once or twice a week  Three or more times a week  **Rice**  Never or occasionally  Once or twice a week  Three or more times a week  **Butter**  Never or occasionally  Once or twice a week  Three or more times a week  **Margarine**  Never or occasionally  Once or twice a week  Three or more times a week  **Nuts**  Never or occasionally  Once or twice a week  Three or more times a week  **Potatoes**  Never or occasionally  Once or twice a week  Three or more times a week  **Milk**  Never or occasionally  Once or twice a week  Three or more times a week  **Eggs**  Never or occasionally  Once or twice a week  Three or more times a week  **Fast food (e.g. burgers)**  Never or occasionally  Once or twice a week  Three or more times a week |
| --- |

**Table S5:** Questions pertaining to health outcomes as presented in the self-administered questionnaire.

| **In the past 12 months, how often, on average, have you taken paracetamol (e.g. Panado)?**  Never  At least once a year  At least once a month |
| --- |
| **In the past 6 months, did you see or talk to a medical doctor or health professional for a respiratory related illness?**  Yes  No |
| **Do you cough a lot?**  Yes  No |
| **If yes, do you cough during the day or night?**  Day  Night |
| **Have you recently woken up in the night because of a cough?**  Yes  No |
| **Have you ever been told that you have asthma?**  Yes  No |
| **Does your cough ever stop you from exercising?**  Yes  No |
| **Does your chest ever sound wheezy or whistling?**  Yes  No |
| **Do you have allergies?**  Yes  No |
| **Do you often have a stuffy, itchy, runny nose or watery, itchy eyes?**  Yes  No |
| **Have you ever been told by a doctor that you have bronchitis?**  Yes  No |
| **Have you ever been told by a doctor that you have pneumonia?**  Yes  No |
| **Have you ever been told by a doctor that you have hay fever?**  Yes  No |
| **Have you ever been told by a doctor that you have TB?**  Yes  No |
| **Have you had any chest injuries?**  Yes  No |
| **Do you have skin problems?**  Yes  No |
| **Do you have other health problems? (You can choose more than 1)**  Arthritis  Heart failure  Stroke  Cataracts  Thyroid disease  Kidney issues  Cancer  Blood pressure  Diabetes  Other |

**Table S6:** Questions pertaining to habits as presented in the self-administered questionnaire.

| **How many times a week do you engage in vigorous physical activity long enough to make you breathe hard?**  Never or occasionally  Once or twice in two weeks  Three or more times a week |
| --- |
| **During a normal week, how many hours a day (24 hours) do you watch television?**  Less than 1 hour  1 hour but less than 3 hours  3 hours but less than 5 hours  5 hours or more |
| **Have you had a regular/daily routine, like regular meals and a regular bedtime?**  Yes  No |
| **Do you smoke?**  Yes  No |
| **I participate in organized religious activities**  Not at all  A little  Not a little and not a lot/in-between/sometimes  Quite a lot  A lot |
| **Do you exercise every day?**  Yes  No |

# Table S7. Overview of air quality data measured at Club Station between 2005 and 2019 in relation to the NAAQS. Grey blocks indicate averaging parameters not available for the respective pollutant; years without data or years that did not pass the data completeness threshold of 75% denoted with “--”; NAAQS exceedances highlighted in bold.

| **Pollutant** | **NAAQS limit** | 2005 | | | | 2006 | | | | 2007 | | | |
| --- | --- | --- | --- | --- | --- | --- | --- | --- | --- | --- | --- | --- | --- |
|  |  | 99^th^ percentile 1-hr average | 99^th^ percentile 8-hr running average | 99^th^ percentile 24-hr average | Annual | 99^th^ percentile 1-hr average | 99^th^ percentile 8-hr running average | 99^th^ percentile 24-hr average | Annual | 99^th^ percentile 1-hr average | 99^th^ percentile 8-hr running average | 99^th^ percentile 24-hr average | Annual |
| PM_2.5_ | 24-hr: 40 µg/m^3^  1 year: 20 µg/m^3^ |  |  | -- | -- |  |  | -- | -- |  |  | -- | -- |
| PM_10_ | 24-hr: 75 µg/m^3^  1 year: 40 µg/m^3^ |  |  | **117** | 38 |  |  | **160** | 36 |  |  | **160** | 39 |
| SO_2_ | 1-hr: 134 ppb  24 hrs: 48 ppb  1 year: 19 ppb | 87 |  | 39 | 9 | 81 |  | 41 | 9 | 116 |  | **51** | 7 |
| NO_2_ | 1-hr: 106 ppb  1 year: 21 ppb | 37 |  |  | 8 | 34 |  |  | -- | **171** |  |  | 11 |
| O_3_ | 61 ppb |  | **83** |  |  |  | **77** |  |  |  | **68** |  |  |
|  | | | | | | | | | | | | | |
| **Pollutant** | **NAAQS limit** | 2008 | | | | 2009 | | | | 2010 | | | |
|  |  | 99^th^ percentile 1-hr average | 99^th^ percentile 8-hr running average | 99^th^ percentile 24-hr average | Annual | 99^th^ percentile 1-hr average | 99^th^ percentile 8-hr running average | 99^th^ percentile 24-hr average | Annual | 99^th^ percentile 1-hr average | 99^th^ percentile 8-hr running average | 99^th^ percentile 24-hr average | Annual |
| PM_2.5_ | 24-hr: 40 µg/m^3^  1 year: 20 µg/m^3^ |  |  | -- | -- |  |  | -- | -- |  |  | -- | -- |
| PM_10_ | 24-hr: 75 µg/m^3^  1 year: 40 µg/m^3^ |  |  | **168** | 37 |  |  | **74** | -- |  |  | **77** | 30 |
| SO_2_ | 1-hr: 134 ppb  24 hrs: 48 ppb  1 year: 19 ppb | 102 |  | 46 | -- | 74 |  | 32 | 8 | 77 |  | 27 | 8 |
| NO_2_ | 1-hr: 106 ppb  1 year: 21 ppb | 92 |  |  | 11 | 39 |  |  | 8 | 51 |  |  | 11 |
| O_3_ | 61 ppb |  | **107** |  |  |  | **76** |  |  |  | **64** |  |  |
|  | | | | | | | | | | | | | |
| **Pollutant** | **NAAQS limit** | 2011 | | | | 2012 | | | | 2013 | | | |
|  |  | 99^th^ percentile 1-hr average | 99^th^ percentile 8-hr running average | 99^th^ percentile 24-hr average | Annual | 99^th^ percentile 1-hr average | 99^th^ percentile 8-hr running average | 99^th^ percentile 24-hr average | Annual | 99^th^ percentile 1-hr average | 99^th^ percentile 8-hr running average | 99^th^ percentile 24-hr average | Annual |
| PM_2.5_ | 24-hr: 40 µg/m^3^  1 year: 20 µg/m^3^ |  |  | 36 | -- |  |  | 30 | 13 |  |  | 37 | 13 |
| PM_10_ | 24-hr: 75 µg/m^3^  1 year: 40 µg/m^3^ |  |  | **90** | 28 |  |  | **75** | 31 |  |  | **98** | 34 |
| SO_2_ | 1-hr: 134 ppb  24 hrs: 48 ppb  1 year: 19 ppb | 82 |  | 35 | 8 | 82 |  | 32 | 8 | 69 |  | 31 | 8 |
| NO_2_ | 1-hr: 106 ppb  1 year: 21 ppb | 54 |  |  | 11 | 42 |  |  | 10 | 51 |  |  | 12 |
| O_3_ | 61 ppb |  | **76** |  |  |  | **71** |  |  |  | **170** |  |  |
|  | | | | | | | | | | | | | |
| **Pollutant** | **NAAQS limit** | 2014 | | | | 2015 | | | | 2016 | | | |
|  |  | 99^th^ percentile 1-hr average | 99^th^ percentile 8-hr running average | 99^th^ percentile 24-hr average | Annual | 99^th^ percentile 1-hr average | 99^th^ percentile 8-hr running average | 99^th^ percentile 24-hr average | Annual | 99^th^ percentile 1-hr average | 99^th^ percentile 8-hr running average | 99^th^ percentile 24-hr average | Annual |
| PM_2.5_ | 24-hr: 40 µg/m^3^  1 year: 20 µg/m^3^ |  |  | **47** | 16 |  |  | **44** | -- |  |  | **59** | 17 |
| PM_10_ | 24-hr: 75 µg/m^3^  1 year: 40 µg/m^3^ |  |  | **151** | **46** |  |  | **84** | -- |  |  | **102** | 39 |
| SO_2_ | 1-hr: 134 ppb  24 hrs: 48 ppb  1 year: 19 ppb | 79 |  | 35 | 10 | 77 |  | 30 | 8 | 71 |  | 32 | 8 |
| NO_2_ | 1-hr: 106 ppb  1 year: 21 ppb | 54 |  |  | 15 | 45 |  |  | 12 | 45 |  |  | 10 |
| O_3_ | 61 ppb |  | **83** |  |  |  | **72** |  |  |  | **200** |  |  |
|  | | | | | | | | | | | | | |
| **Pollutant** | **NAAQS limit** | 2017 | | | | 2018 | | | | 2019 | | | |
|  |  | 99^th^ percentile 1-hr average | 99^th^ percentile 8-hr running average | 99^th^ percentile 24-hr average | Annual | 99^th^ percentile 1-hr average | 99^th^ percentile 8-hr running average | 99^th^ percentile 24-hr average | Annual | 99^th^ percentile 1-hr average | 99^th^ percentile 8-hr running average | 99^th^ percentile 24-hr average | Annual |
| PM_2.5_ | 24-hr: 40 µg/m^3^  1 year: 20 µg/m^3^ |  |  | 27 | -- |  |  | -- | -- |  |  | **99** | -- |
| PM_10_ | 24-hr: 75 µg/m^3^  1 year: 40 µg/m^3^ |  |  | 32 | -- |  |  | -- | -- |  |  | 54 | -- |
| SO_2_ | 1-hr: 134 ppb  24 hrs: 48 ppb  1 year: 19 ppb | **62** |  | 9 | -- | -- |  | -- | -- | 79 |  | 31 | -- |
| NO_2_ | 1-hr: 106 ppb  1 year: 21 ppb | 39 |  |  | -- | -- |  |  | -- | 39 |  |  | -- |
| O_3_ | 61 ppb |  | **63** |  |  |  | -- |  |  |  | **71** |  |  |

Note. NAAQS: National Ambient Air Quality Standard.

# Table S8. Overview of air quality data measured at Embalenhle SAWS station between 2005 and 2019 in relation to the NAAQS. Grey blocks indicate averaging parameters not available for the respective pollutant; years without data or years that did not pass the data completeness threshold of 75% denoted with “--”; NAAQS exceedances highlighted in bold.

| **Pollutant** | **NAAQS limit** | 2005 | | | | 2006 | | | | 2007 | | | |
| --- | --- | --- | --- | --- | --- | --- | --- | --- | --- | --- | --- | --- | --- |
|  |  | 99^th^ percentile 1-hr average | 99^th^ percentile 8-hr running average | 99^th^ percentile 24-hr average | Annual | 99^th^ percentile 1-hr average | 99^th^ percentile 8-hr running average | 99^th^ percentile 24-hr average | Annual | 99^th^ percentile 1-hr average | 99^th^ percentile 8-hr running average | 99^th^ percentile 24-hr average | Annual |
| PM_2.5_ | 24-hr: 40 µg/m^3^  1 year: 20 µg/m^3^ |  |  | -- | -- |  |  | -- | -- |  |  | -- | -- |
| PM_10_ | 24-hr: 75 µg/m^3^  1 year: 40 µg/m^3^ |  |  | **--** | -- |  |  | **--** | -- |  |  | **--** | -- |
| SO_2_ | 1-hr: 134 ppb  24 hrs: 48 ppb  1 year: 19 ppb | -- |  | -- | -- | -- |  | -- | -- | -- |  | -- | -- |
| NO_2_ | 1-hr: 106 ppb  1 year: 21 ppb | -- |  |  | -- | -- |  |  | -- | -- |  |  | -- |
| O_3_ | 61 ppb |  | -- |  |  |  | -- |  |  |  | -- |  |  |
|  | | | | | | | | | | | | | |
| **Pollutant** | **NAAQS limit** | 2008 | | | | 2009 | | | | 2010 | | | |
|  |  | 99^th^ percentile 1-hr average | 99^th^ percentile 8-hr running average | 99^th^ percentile 24-hr average | Annual | 99^th^ percentile 1-hr average | 99^th^ percentile 8-hr running average | 99^th^ percentile 24-hr average | Annual | 99^th^ percentile 1-hr average | 99^th^ percentile 8-hr running average | 99^th^ percentile 24-hr average | Annual |
| PM_2.5_ | 24-hr: 40 µg/m^3^  1 year: 20 µg/m^3^ |  |  | **124** | -- |  |  | **149** | **39** |  |  | **149** | **38** |
| PM_10_ | 24-hr: 75 µg/m^3^  1 year: 40 µg/m^3^ |  |  | **310** | -- |  |  | **275** | **73** |  |  | **346** | **88** |
| SO_2_ | 1-hr: 134 ppb  24 hrs: 48 ppb  1 year: 19 ppb | 28 |  | 16 | -- | 52 |  | 31 | 9 | 61 |  | 30 | 11 |
| NO_2_ | 1-hr: 106 ppb  1 year: 21 ppb | 72 |  |  | -- | 59 |  |  | -- | 71 |  |  | 20 |
| O_3_ | 61 ppb |  | **--** |  |  |  | -- |  |  |  | -- |  |  |
|  | | | | | | | | | | | | | |
| **Pollutant** | **NAAQS limit** | 2011 | | | | 2012 | | | | 2013 | | | |
|  |  | 99^th^ percentile 1-hr average | 99^th^ percentile 8-hr running average | 99^th^ percentile 24-hr average | Annual | 99^th^ percentile 1-hr average | 99^th^ percentile 8-hr running average | 99^th^ percentile 24-hr average | Annual | 99^th^ percentile 1-hr average | 99^th^ percentile 8-hr running average | 99^th^ percentile 24-hr average | Annual |
| PM_2.5_ | 24-hr: 40 µg/m^3^  1 year: 20 µg/m^3^ |  |  | **204** | -- |  |  | **134** | **28** |  |  | **112** | **29** |
| PM_10_ | 24-hr: 75 µg/m^3^  1 year: 40 µg/m^3^ |  |  | **371** | -- |  |  | **270** | **61** |  |  | **320** | **72** |
| SO_2_ | 1-hr: 134 ppb  24 hrs: 48 ppb  1 year: 19 ppb | 54 |  | 44 | -- | 57 |  | **54** | 10 | 64 |  | 38 | -- |
| NO_2_ | 1-hr: 106 ppb  1 year: 21 ppb | 32 |  |  | -- | 35 |  |  | 7 | 37 |  |  | 12 |
| O_3_ | 61 ppb |  | -- |  |  |  | -- |  |  |  | -- |  |  |
|  | | | | | | | | | | | | | |
| **Pollutant** | **NAAQS limit** | 2014 | | | | 2015 | | | | 2016 | | | |
|  |  | 99^th^ percentile 1-hr average | 99^th^ percentile 8-hr running average | 99^th^ percentile 24-hr average | Annual | 99^th^ percentile 1-hr average | 99^th^ percentile 8-hr running average | 99^th^ percentile 24-hr average | Annual | 99^th^ percentile 1-hr average | 99^th^ percentile 8-hr running average | 99^th^ percentile 24-hr average | Annual |
| PM_2.5_ | 24-hr: 40 µg/m^3^  1 year: 20 µg/m^3^ |  |  | **115** | -- |  |  | **46** | -- |  |  | **136** | -- |
| PM_10_ | 24-hr: 75 µg/m^3^  1 year: 40 µg/m^3^ |  |  | **229** | -- |  |  | **112** | -- |  |  | **268** | -- |
| SO_2_ | 1-hr: 134 ppb  24 hrs: 48 ppb  1 year: 19 ppb | 75 |  | 37 | 11 | 48 |  | 24 | -- | 55 |  | 27 | 7 |
| NO_2_ | 1-hr: 106 ppb  1 year: 21 ppb | 41 |  |  | 15 | 82 |  |  | -- | 91 |  |  | -- |
| O_3_ | 61 ppb |  | -- |  |  |  | -- |  |  |  | -- |  |  |
|  | | | | | | | | | | | | | |
| **Pollutant** | **NAAQS limit** | 2017 | | | | 2018 | | | | 2019 | | | |
|  |  | 99^th^ percentile 1-hr average | 99^th^ percentile 8-hr running average | 99^th^ percentile 24-hr average | Annual | 99^th^ percentile 1-hr average | 99^th^ percentile 8-hr running average | 99^th^ percentile 24-hr average | Annual | 99^th^ percentile 1-hr average | 99^th^ percentile 8-hr running average | 99^th^ percentile 24-hr average | Annual |
| PM_2.5_ | 24-hr: 40 µg/m^3^  1 year: 20 µg/m^3^ |  |  | **115** | -- |  |  | -- | -- |  |  | **--** | -- |
| PM_10_ | 24-hr: 75 µg/m^3^  1 year: 40 µg/m^3^ |  |  | **214** | -- |  |  | 54 | -- |  |  | **--** | -- |
| SO_2_ | 1-hr: 134 ppb  24 hrs: 48 ppb  1 year: 19 ppb | 38 |  | 17 | 5 | 13 |  | 5 | 2 | 40 |  | 16 | 4 |
| NO_2_ | 1-hr: 106 ppb  1 year: 21 ppb | 137 |  |  | -- | **111** |  |  | -- | 40 |  |  | -- |
| O_3_ | 61 ppb |  | -- |  |  |  | --- |  |  |  | -- |  |  |

Note. NAAQS: National Ambient Air Quality Standard.

# Table S9. Overview of air quality data measured at Embalenhle Sasol station between 2005 and 2019 in relation to the NAAQS. Grey blocks indicate averaging parameters not available for the respective pollutant; years without data or years that did not pass the data completeness threshold of 75% denoted with “--”; NAAQS exceedances highlighted in bold.

| **Pollutant** | **NAAQS limit** | 2005 | | | | 2006 | | | | 2007 | | | |
| --- | --- | --- | --- | --- | --- | --- | --- | --- | --- | --- | --- | --- | --- |
|  |  | 99^th^ percentile 1-hr average | 99^th^ percentile 8-hr running average | 99^th^ percentile 24-hr average | Annual | 99^th^ percentile 1-hr average | 99^th^ percentile 8-hr running average | 99^th^ percentile 24-hr average | Annual | 99^th^ percentile 1-hr average | 99^th^ percentile 8-hr running average | 99^th^ percentile 24-hr average | Annual |
| PM_2.5_ | 24-hr: 40 µg/m^3^  1 year: 20 µg/m^3^ |  |  | -- | -- |  |  | -- | -- |  |  | -- | -- |
| PM_10_ | 24-hr: 75 µg/m^3^  1 year: 40 µg/m^3^ |  |  | **--** | **--** |  |  | **--** | **--** |  |  | **--** | **--** |
| SO_2_ | 1-hr: 134 ppb  24 hrs: 48 ppb  1 year: 19 ppb | -- |  | **--** | **--** |  |  | **--** | **--** | -- |  | **--** | **--** |
| NO_2_ | 1-hr: 106 ppb  1 year: 21 ppb | -- |  |  | **--** | **--** |  |  | **--** | **--** |  |  | -- |
| O_3_ | 61 ppb |  | -- |  |  |  | -- |  |  |  | -- |  |  |
|  | | | | | | | | | | | | | |
| **Pollutant** | **NAAQS limit** | 2008 | | | | 2009 | | | | 2010 | | | |
|  |  | 99^th^ percentile 1-hr average | 99^th^ percentile 8-hr running average | 99^th^ percentile 24-hr average | Annual | 99^th^ percentile 1-hr average | 99^th^ percentile 8-hr running average | 99^th^ percentile 24-hr average | Annual | 99^th^ percentile 1-hr average | 99^th^ percentile 8-hr running average | 99^th^ percentile 24-hr average | Annual |
| PM_2.5_ | 24-hr: 40 µg/m^3^  1 year: 20 µg/m^3^ |  |  | -- | -- |  |  | -- | -- |  |  | -- | -- |
| PM_10_ | 24-hr: 75 µg/m^3^  1 year: 40 µg/m^3^ |  |  | **--** | **--** |  |  | **--** | **--** |  |  | **--** | **--** |
| SO_2_ | 1-hr: 134 ppb  24 hrs: 48 ppb  1 year: 19 ppb | -- |  | **--** | **--** | -- |  | **--** | **--** | -- |  | **--** | **--** |
| NO_2_ | 1-hr: 106 ppb  1 year: 21 ppb | -- |  |  | **--** | **--** |  |  | **--** | **--** |  |  | -- |
| O_3_ | 61 ppb |  | **--** |  |  |  | -- |  |  |  | -- |  |  |
|  | | | | | | | | | | | | | |
| **Pollutant** | **NAAQS limit** | 2011 | | | | 2012 | | | | 2013 | | | |
|  |  | 99^th^ percentile 1-hr average | 99^th^ percentile 8-hr running average | 99^th^ percentile 24-hr average | Annual | 99^th^ percentile 1-hr average | 99^th^ percentile 8-hr running average | 99^th^ percentile 24-hr average | Annual | 99^th^ percentile 1-hr average | 99^th^ percentile 8-hr running average | 99^th^ percentile 24-hr average | Annual |
| PM_2.5_ | 24-hr: 40 µg/m^3^  1 year: 20 µg/m^3^ |  |  | -- | -- |  |  | -- | -- |  |  | *--* | *--* |
| PM_10_ | 24-hr: 75 µg/m^3^  1 year: 40 µg/m^3^ |  |  | **--** | **--** |  |  | **--** | **--** |  |  | ***--*** | ***--*** |
| SO_2_ | 1-hr: 134 ppb  24 hrs: 48 ppb  1 year: 19 ppb | -- |  | **--** | **--** | -- |  | -- | **--** | **--** |  | **--** | **--** |
| NO_2_ | 1-hr: 106 ppb  1 year: 21 ppb | -- |  |  | **--** | **--** |  |  | **--** | **--** |  |  | -- |
| O_3_ | 61 ppb |  | -- |  |  |  | -- |  |  |  | -- |  |  |
|  | | | | | | | | | | | | | |
| **Pollutant** | **NAAQS limit** | 2014 | | | | 2015 | | | | 2016 | | | |
|  |  | 99^th^ percentile 1-hr average | 99^th^ percentile 8-hr running average | 99^th^ percentile 24-hr average | Annual | 99^th^ percentile 1-hr average | 99^th^ percentile 8-hr running average | 99^th^ percentile 24-hr average | Annual | 99^th^ percentile 1-hr average | 99^th^ percentile 8-hr running average | 99^th^ percentile 24-hr average | Annual |
| PM_2.5_ | 24-hr: 40 µg/m^3^  1 year: 20 µg/m^3^ |  |  | -- | -- |  |  | -- | -- |  |  | **67** | **21** |
| PM_10_ | 24-hr: 75 µg/m^3^  1 year: 40 µg/m^3^ |  |  | **--** | **--** |  |  | **--** | **--** |  |  | **144** | **51** |
| SO_2_ | 1-hr: 134 ppb  24 hrs: 48 ppb  1 year: 19 ppb | -- |  | **--** | **--** | -- |  | **--** | **--** | 72 |  | 28 | 8 |
| NO_2_ | 1-hr: 106 ppb  1 year: 21 ppb | -- |  |  | -- | -- |  |  | -- | **110** | -- |  | -- |
| O_3_ | 61 ppb |  | -- |  |  |  | -- |  |  |  | **71** |  |  |
|  | | | | | | | | | | | | | |
| **Pollutant** | **NAAQS limit** | 2017 | | | | 2018 | | | | 2019 | | | |
|  |  | 99^th^ percentile 1-hr average | 99^th^ percentile 8-hr running average | 99^th^ percentile 24-hr average | Annual | 99^th^ percentile 1-hr average | 99^th^ percentile 8-hr running average | 99^th^ percentile 24-hr average | Annual | 99^th^ percentile 1-hr average | 99^th^ percentile 8-hr running average | 99^th^ percentile 24-hr average | Annual |
| PM_2.5_ | 24-hr: 40 µg/m^3^  1 year: 20 µg/m^3^ |  |  | **57** | **21** |  |  | **55** | -- |  |  | **41** | -- |
| PM_10_ | 24-hr: 75 µg/m^3^  1 year: 40 µg/m^3^ |  |  | **150** | **53** |  |  | **122** | -- |  |  | 70 | -- |
| SO_2_ | 1-hr: 134 ppb  24 hrs: 48 ppb  1 year: 19 ppb | 63 |  | 26 | 7 | 68 |  | 31 | -- | 61 |  | 24 | -- |
| NO_2_ | 1-hr: 106 ppb  1 year: 21 ppb | 40 |  |  | 12 | 41 |  | -- |  | 36 |  |  | -- |
| O_3_ | 61 ppb |  | **71** |  |  |  | **68** |  |  |  | **73** |  |  |

Note. NAAQS: National Ambient Air Quality Standard.
